# Regulation of doxorubicin resistance and cellular metabolism by miR-203a-3p via p53 and TAp63 signaling in hepatocellular carcinoma

**DOI:** 10.3389/fphar.2026.1803189

**Published:** 2026-06-12

**Authors:** Noha M. Abdelaal, Anwar Abdelnaser

**Affiliations:** 1 Biotechnology Graduate Program, School of Sciences and Engineering, The American University in Cairo, New Cairo, Egypt; 2 Institute of Global Health and Human Ecology, School of Sciences and Engineering, The American University in Cairo, New Cairo, Egypt

**Keywords:** apoptosis, doxorubicin resistance, metabolic rewiring, MiR-203a-3p, p53 signaling, TAp63 signaling

## Abstract

Doxorubicin (DOX) is a cornerstone chemotherapeutic drug in the treatment of hepatocellular carcinoma (HCC). However, its efficacy is often limited by the development of drug resistance linked to increased cellular capacity to repair DNA damage. Altered tumor metabolism allows cancer cells to meet increased energy demands for rapid proliferation while evading apoptosis and adapting to therapeutic interventions. MiR-203a-3p is associated with regulating members of the p53 family and has been implicated in regulating chemoresistance and metabolic rewiring in various cancers, yet its role in HCC remains to be elucidated. This study investigated the functional role of miR-203a-3p in response to DOX in HCC cell lines differing in p53 status. HepG2 (wild-type p53) and Huh7 (mutant p53) cells were transfected with miR-203a-3p mimics or inhibitors, alone or in combination with DOX. Cell viability was assessed by MTT assay, and the expression levels of p53 family members and Bax were measured by qPCR. Apoptosis was evaluated by flow cytometry, and mitochondrial function was examined using the Seahorse XFe96 analyzer. MiR-203a-3p expression was significantly higher in DOX-resistant HepG2 cells relative to DOX-sensitive Huh7 cells. In HepG2 cells, miR-203a-3p promoted resistance through p53/Δ133p53-driven survival and enhanced oxidative phosphorylation. In Huh7 cells, it suppressed TAp63/Bax-mediated apoptosis while driving both oxidative phosphorylation and glycolysis, promoting resistance despite the absence of wild-type p53. These findings identify miR-203a-3p as a key modulator of DOX resistance in HCC through coordinated regulation of p53 family expression, apoptotic signaling, and metabolic rewiring, highlighting its potential as a therapeutic target for miRNA-based combination therapies.

## Graphical Abstract

**Figure:**
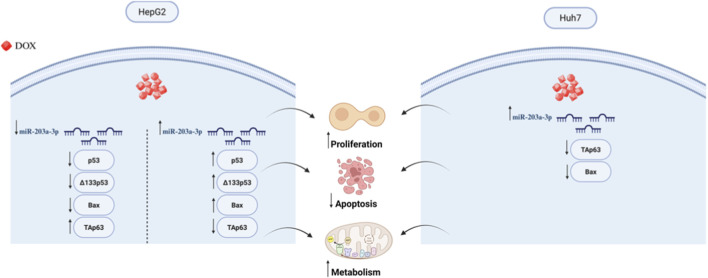

## 1 Introduction

Liver cancer is a significant global health concern and ranks third in mortality rates among all cancers (Bray et al., 2024). Hepatocellular carcinoma (HCC), which accounts for 80%–90% of primary liver cancers, is an aggressive malignancy that primarily develops on the background of chronic liver disease and shows recurrence rates of up to 88% (Mejia and Pasko, 2020; Tsilimigras et al., 2020). Doxorubicin (DOX) is a widely used anthracycline antibiotic for HCC (Kciuk et al., 2023). By inhibiting topoisomerase II, DOX prevents the re-ligation of double-strand breaks, leading to persistent DNA damage, p53-dependent cell cycle arrest, and apoptosis (van der Zanden et al., 2021). However, DOX resistance remains a critical clinical challenge. Mechanisms implicated include alterations in drug efflux transporters, mutations in drug targets, enhanced DNA repair, and inhibition of apoptosis, though these processes remain to be fully characterized (Micallef and Baron, 2020; Sritharan and Sivalingam, 2021).

The p53 family of transcription factors is central to cellular responses to chemotherapy, with implications for both therapeutic efficacy and mechanisms of resistance (Abuetabh et al., 2022). p53 regulates the expression of over 500 genes and is mutated in 25%–50% of HCC patients (Bieging et al., 2014; Kunst et al., 2016). HCCs that retain wild-type p53 commonly exhibit disruptions in the p53-mediated apoptotic pathway (Choudhary et al., 2023). p53 activation in hepatocytes primarily induces cell-cycle arrest while suppressing mitochondrial-dependent apoptosis. One proposed mechanism underlying this shift involves p53-driven upregulation of hepatic insulin-like growth factor-binding protein-1 (IGFBP1), which antagonizes mitochondrial p53 signaling and inhibits apoptosis (Leu and George, 2007; Xue et al., 2007). The complexity of the p53 pathway has increased following the recent discovery of 12 p53 isoforms, including Δ133p53, which exhibits oncogenic functions and correlates with poor prognosis (Anbarasan and Bourdon, 2019; Joruiz et al., 2020; Steffens Reinhardt et al., 2023).

The p63 gene shares high structural homology with p53 and produces two opposing isoforms: the full-length transcriptionally active TAp63, which acts as a tumor suppressor, and the dominant-negative ΔNp63, which displays oncogenic activity (Pokorná et al., 2021). In HCC, p63 has emerged as a functionally significant member of the p53 family. A molecular profile characterized by reduced TAp63 expression together with elevated ΔNp63 levels in HCC patients is associated with higher rates of tumor recurrence and reduced survival (González et al., 2017). TAp63 promotes apoptosis by upregulating pro-apoptotic genes, including the Bcl-2 family members Bax and Puma, and death receptors CD95 and TRAIL-R, thereby activating both intrinsic and extrinsic pathways (Komatsu et al., 2009; Gressner et al., 2005). Additionally, its expression correlates with increased sensitivity to chemotherapeutic drugs bleomycin, cisplatin, and DOX (Gressner et al., 2005).

MicroRNAs (miRNAs), small non-coding RNA molecules, negatively regulate gene expression post-transcriptionally by binding to the 3′untranslated region (3′UTR) of target mRNAs (Eulalio et al., 2008). Recent evidence has also revealed that miRNAs act as positive regulators of gene expression, proposing several potential mechanisms (Tan et al., 2019). They play central roles in key biological processes and cancer hallmarks, and have emerged as critical regulators of chemoresistance (Mondal and Meeran, 2021; Slack and Chinnaiyan, 2019). MicroRNA-203a-3p (miR-203a-3p), the first identified skin-specific miRNA, is frequently dysregulated in cancer (Sonkoly et al., 2008). By regulating pathways involved in proliferation, apoptosis, metastasis, stemness, and metabolism, it influences multiple cancer hallmarks (Chang et al., 2017; Michel and Malumbres, 2013). MiR-203a-3p has also been implicated in regulating chemotherapeutic drug resistance in several cancers including oral squamous cell carcinoma and osteosarcoma (Biswal and Mallick, 2024; Huang et al., 2021). In HCC, contradictory findings have been reported. While some studies suggest miR-203a-3p acts as a tumor suppressor by inhibiting angiogenesis and inducing apoptosis, other findings indicate a role in promoting cell survival and metastasis (Guo et al., 2023; Huo et al., 2017; Wang et al., 2018). Its role in modulating chemoresistance in HCC remains to be elucidated.

Computational predictions have identified p53 as a potential target of miR-203a-3p, while experimental validation confirms targeting of another member of the p53 family, ΔNp63 (Vejnar and Zdobnov, 2012; Yi et al., 2008). Both ΔNp63 and TAp63 isoforms share the same 3′UTR of the p63 gene, suggesting a potential miR-203a-3p regulatory mechanism involving multiple p53 family members (Bui et al., 2020). We hypothesized that miR-203a-3p modulates DOX resistance by regulating the expression of p53 family members. This study therefore investigated the role of miR-203a-3p in modulating DOX resistance in HCC cell lines differing in p53 status: HepG2 (wild-type) and Huh7 (mutant). We further examined the involvement of Δ133p53, TAp63, and Bax, a shared transcriptional target of p53 and TAp63 (Hernández Borrero and El-Deiry, 2021; Yin et al., 2021). Additionally, we evaluated the effects of miR-203a-3p modulation on apoptotic signaling and cellular metabolism.

## 2 Materials and methods

### 2.1 Cell culture

HepG2 cell line (ATCC, Cat# HB-8065, RRID: CVCL_0027) was obtained from Children’s Cancer Hospital 57357 (Egypt). Huh7 cell line (JCRB, Cat# 0403, RRID: CVCL_0336) was obtained from Nawah Scientific (Egypt). Cells were cultured in high-glucose Dulbecco’s Modified Eagle Medium (DMEM; Capricorn Scientific, Germany) supplemented with 10% Fetal bovine serum (FBS; Capricorn Scientific, Germany) and 1% Penicillin-Streptomycin (Pen-Strep; Thermo Fisher Scientific, United States), in a humidified incubator at 37 °C with 5% CO_2_ and were sub-cultured at 70%–80% confluency.

### 2.2 DOX preparation and oligonucleotide transfection

Stock solutions of doxorubicin hydrochloride (Tocris Bioscience, UK) were prepared in DMSO and diluted in serum-free medium to the desired concentrations, with the final DMSO concentration maintained below 0.1%. HepG2 cells were transiently transfected with 50 nM hsa-miR-203a-3p miRCURY LNA miRNA mimic, 100 nM inhibitor, or 100 nM AllStars siRNA negative control (NC) (Qiagen, Germany). Huh7 cells were transfected with 30 nM mimic or negative control. Forward transfection was carried out using HiPerFect transfection reagent (Qiagen, Germany) in serum-free medium, according to the manufacturer’s instructions. Cells were collected 24 h post-transfection for analysis.

### 2.3 Cell viability assay

Cells were seeded in 96-well plates at 1.5 × 10^4^ cells/well and treated with increasing DOX concentrations (0.05–5 µM) for 6 and 24 h. Cells were then incubated with 5 mg/mL MTT solution for 2 h (Thermo Fisher Scientific, United States), after which formazan crystals were dissolved in DMSO and absorbance was measured at 570 nm on the Infinite 200 Pro microplate reader (Tecan, Switzerland). IC_50_ values were calculated from normalized dose-response curves, and 24 h IC_50_ concentrations were used for subsequent experiments. In combination assays, cells were seeded as above, transfected with miR-203a-3p mimic, inhibitor, or negative control, and treated with DOX (0.05–5 µM) for 24 h. Cell viability was then measured by MTT assay.

### 2.4 Real-time PCR

Cells were seeded in 24-well plates at 1 × 10^5^ cells/well. For DOX treatment, cells were exposed to DOX at IC_50_ concentrations for 6 h before RT-qPCR. For transfection, cells were transfected with miR-203a-3p mimic, inhibitor, or negative control for 24 h and processed for RT-qPCR. In combination assays, cells were transfected as above, then treated with DOX IC_50_ concentrations for 6 h before RT-qPCR. Total RNA was isolated using Qiazol (Qiagen, Germany). MiR-203a-3p was purified with the miRNeasy Mini Kit (Qiagen, Germany). RNA purity and concentration were determined using NanoPhotometer N60 (Implen, Germany). For mRNA, cDNA was prepared from 1 μg of total RNA using RevertAid First Strand cDNA Synthesis Kit (Thermo Fisher Scientific, United States), and gene expression was quantified using PowerUp SYBR Green Master Mix (Thermo Fisher Scientific, United States). β-Actin functioned as the internal control. Primer sequences are listed in Supplementary Table S1. For miR-203a-3p, cDNA was prepared from 30 ng total RNA using the miRCURY LNA RT Kit (Qiagen, Germany), and miR-203a-3p expression was quantified using miRCURY LNA miRNA SYBR Green Master Mix (Qiagen, Germany). Based on the manufacturer’s recommendation, hsa-miR-103a-3p served as the internal control, with primer sequences listed in Supplementary Table S2. qPCR was performed on a CFX96 Real-Time PCR Detection System (Bio-Rad, United States). Gene expression levels were calculated using the 2^−ΔΔ*C*^ ^T^ method.

### 2.5 Flow cytometry analysis

Apoptosis was assessed using Annexin V/fluorescein isothiocyanate (FITC) and propidium iodide (PI) apoptosis detection kit (Elabscience, China). Cells were seeded in 24-well plates at 1 × 10^5^ cells/well, transfected with miR-203a-3p mimic, inhibitor, or negative control, and in combination assays treated with DOX IC_50_ concentrations for 24 h. Briefly, cells were collected, washed with ice-cold PBS, resuspended in 500 μL of 1x Annexin V Binding Buffer, and stained with 5 µL Annexin V-FITC and 5 µL PI for 15–20 min at room temperature in the dark. Apoptosis was analyzed on an Attune NxT cytometer (Thermo Fisher Scientific, United States), with 30,000 events/sample acquired. Cells were classified as viable (Annexin V-/PI-), early apoptotic (Annexin V+/PI-), late apoptotic (Annexin V+/PI+), or necrotic (Annexin V-/PI+). Data were analyzed using FlowJo software v10.10 and expressed as the percentage of cells positive for early and late apoptosis.

### 2.6 Seahorse cell mitochondrial stress test

The oxygen consumption rate (OCR) and extracellular acidification rate (ECAR) of cells in real time were measured using the Seahorse XF Cell Mito Stress Test using the Seahorse XFe96 analyzer (Agilent Technologies, United States). HepG2 and Huh7 cells were seeded in Seahorse XF96 cell culture microplates at 4 × 10^3^ and 1.2 × 10^4^ cells/80 µL/well, respectively, transfected with miR-203a-3p mimic, inhibitor, or negative control, and in combination assays treated with DOX IC_50_ concentrations for 24 h. A Seahorse XF96 sensor cartridge for each microplate was hydrated in Seahorse XF Calibrant at 37 °C in a non-CO_2_ incubator overnight. Briefly, cells were washed twice with Seahorse XF assay medium supplemented with 10 mM Seahorse XF glucose, 1 mM Seahorse XF pyruvate, and 2 mM Seahorse XF L-glutamine. Following the second wash, cells were incubated with 180 µL/well assay medium in a non-CO_2_ incubator at 37 °C for 1 h. The sensor cartridge injection ports were loaded with oligomycin (3µM, port A), FCCP (1 μM, port B), rotenone/antimycin A (0.5 µM, port C) (Sigma-Aldrich, United States), and assay medium (port D). After calibration, the cell culture microplate was inserted, and OCR and ECAR were recorded in 3-min mix/3-min measure cycles. Data were analyzed using Wave software.

### 2.7 Statistical analysis

Experiments were performed in triplicate. Data are presented as mean ± standard error of the mean (SEM). Statistical analysis was performed using GraphPad Prism 10.4.2. Comparisons between two or more groups were carried out using Student’s t-test or one-way ANOVA followed by Tukey’s multiple comparisons test. Statistical significance is defined as *P < 0.05, **P < 0.01, ***P < 0.001, and ****P < 0.0001.

## 3 Results

### 3.1 Elevated miR-203a-3p levels correlate with increased DOX resistance in HCC cells

**FIGURE 1:**
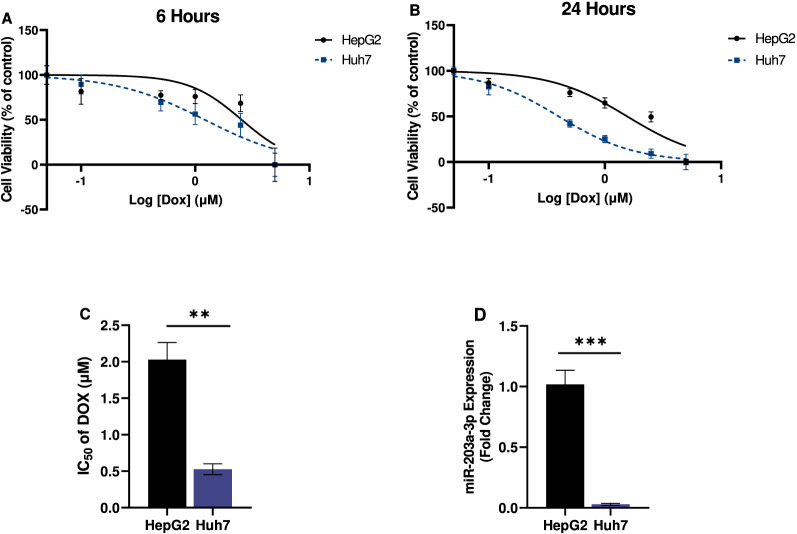
Elevated miR-203a-3p levels correlate with increased DOX resistance in HCC cells. MTT assay showing viability of HCC cells following DOX treatment (0.05, 0.1, 0.5, 1, 2.5, and 5 µM) for 6 h **(A)** and 24 h **(B)**. Data are expressed as % of control (100%) ± SEM (n = 6). Statistical significance was calculated by one-way ANOVA followed by Tukey’s multiple comparisons test. IC_50_ values were calculated based on MTT results following 24 h of DOX treatment **(C)**. Detection of miR-203a-3p expression in HCC cells; the results were quantified by comparing to reference miRNA miR-103a-3p **(D)**. Data are expressed as mean ± SEM (n = 4). Student’s t-test calculated statistical significance. **P < 0.01, ***P < 0.001.

MTT assay revealed that the cell viability of both HCC cell lines was inhibited by DOX in a dose-dependent manner (Figures 1A,B). At 6 h, the IC_50_ values were 2.4 ± 0.7 µM for HepG2 and 1.2 ± 0.6 µM for Huh7, indicating that HepG2 cells were more resistant to DOX treatment compared to Huh7 cells. By 24 h, the IC_50_ values decreased 0.83-fold for HepG2 and 0.42-fold for Huh7, further confirming the significantly higher resistance of HepG2 cells to DOX treatment (Table1; Figure 1C). To explore the potential mechanism underlying this resistance, we examined the expression of miR-203a-3p in both cell lines using qPCR. As shown in Figure 1D, miR-203a-3p was 97% lower in Huh7 cells compared to HepG2 cells. Together, these results highlight the association between elevated miR-203a-3p expression and increased DOX resistance.

**TABLE 1:** IC_50_ values (µM) of DOX in HepG2 and Huh7 cells at specified time intervals.

Time	HepG2	Huh7
6 h	2.4 ± 0.7	1.2 ± 0.6
24 h	2 ± 0.3	0.5 ± 0.1

### 3.2 Altered miR-203a-3p levels modulate DOX resistance in HCC cells

**FIGURE 2:**
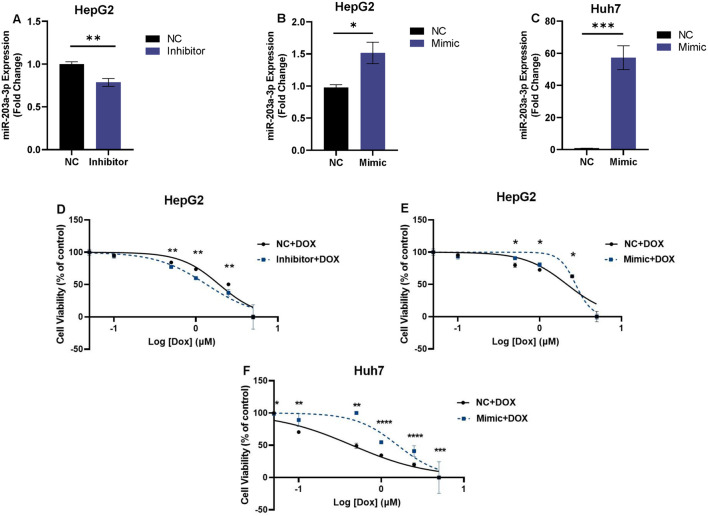
Altered miR-203a-3p levels modulate DOX resistance in HCC cells. MiR-203a-3p levels were measured in HepG2 cells transfected with 100 nM miR-203a-3p inhibitor **(A)** and 50 nM miR-203a-3p mimic **(B)** or 100 nM NC and Huh7 cells transfected with 30 nM miR-203a-3p mimic or NC **(C)**. The results were quantified by comparing to the reference miRNA miR-103a-3p. Data are expressed as mean ± SEM (n = 4). MTT assay showing viability of transfected HepG2 cells **(D,E)** and the viability of transfected Huh7 cells **(F)** following treatment with increasing concentrations of DOX (0.05, 0.1, 0.5, 1, 2.5, and 5 µM) for 24 h. Data are expressed as % of control (100%) ± SEM (n = 3). Statistical significance was calculated using Student’s t-test. *P < 0.05, **P < 0.01, ***P < 0.001, ****P < 0.0001 vs NC.

To assess the functional impact of miR-203a-3p in mediating DOX resistance, we first altered its expression levels in both HCC cell lines. As shown in Figures 2A,B, HepG2 transfection with the inhibitor resulted in a 0.8-fold downregulation of miR-203a-3p, while the mimic induced a 1.52-fold upregulation relative to the negative control. In Huh7 cells, transfection with the mimic led to a 57-fold upregulation of miR-203a-3p, compared to the negative control (Figure 2C). Next, transfected HepG2 and Huh7 cells were treated with increasing concentrations of DOX for 24 h, and cell viability was assessed using the MTT assay. Inhibition of miR-203a-3p significantly increased the sensitivity of HepG2 cells to DOX, resulting in a 0.65-fold reduction in the IC_50_ value, compared to the negative control. In contrast, overexpression of miR-203a-3p conferred significantly greater resistance to DOX, as shown by a 1.4-fold increase in IC_50_ value in HepG2 cells and a 3.2-fold increase in Huh7 cells, relative to their respective negative controls (Table 2; Figures 2D–F). These findings demonstrate that altering miR-203a-3p expression significantly affects DOX resistance in HCC cells.

**TABLE 2:** IC_50_ values (µM) of DOX in HepG2 and Huh7 cells following miR-203a-3p modulation.

Transfection condition	HepG2	Huh7
Negative control + DOX	2 ± 0.5	0.5 ± 0.1
MiR-203a-3p Inhibitor + DOX	1.3 ± 0.4	-
MiR-203a-3p Mimic + DOX	2.8 ± 0.5	1.6 ± 0.7

### 3.3 MiR-203a-3p modulates DOX resistance through the p53 family in HepG2 cells

**FIGURE 3:**
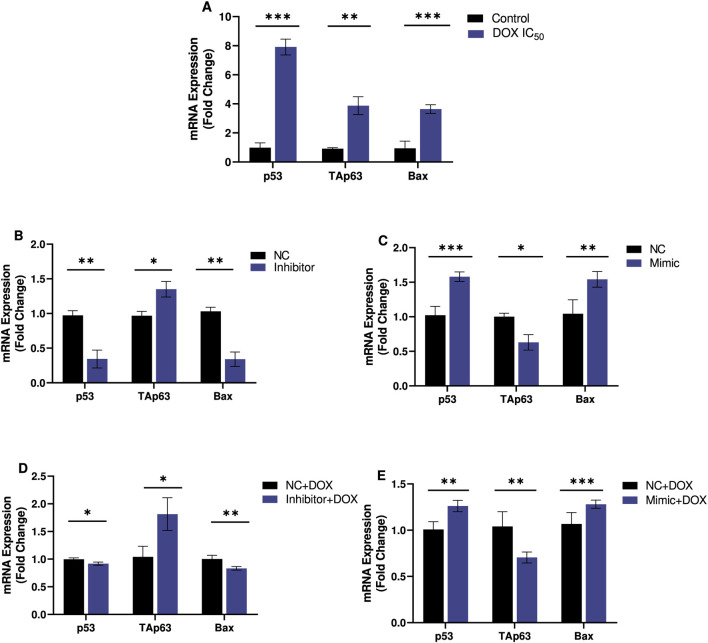
MiR-203a-3p modulates DOX resistance through the p53 family in HepG2 cells. Effect of 2 µM of DOX **(A)**, 100 nM inhibitor **(B)**, 50 nM mimic **(C)**, and combinations **(D,E)** on p53, TAp63, and Bax expression in HepG2 cells detected by qPCR. The results were quantified by comparing to the β-actin reference gene. Data are expressed as mean ± SEM (n = 4). Statistical significance was calculated using Student’s t-test. *P < 0.05, **P < 0.01, ***P < 0.001 vs control, NC, and NC + DOX.

The p53 family plays an essential role in regulating chemoresistance in cancer. To assess its role in miR-203a-3p–mediated DOX resistance in HepG2 cells, qPCR was used to measure p53, TAp63, and their target Bax. As shown in Figures 3A–E, DOX increased p53 expression 7.91-fold, whereas the inhibitor reduced it by 0.34-fold and the mimic increased it by 1.58-fold, relative to their respective controls. In combination assays, the inhibitor downregulated p53 expression by 0.92-fold and the mimic upregulated it by 1.26-fold, relative to the DOX-treated negative control. TAp63 was upregulated 3.88-fold by DOX and 1.35-fold by the inhibitor alone but suppressed 0.63-fold by the mimic. With DOX, the inhibitor further enhanced TAp63 by 1.81-fold, while the mimic attenuated it by 0.71-fold. Despite being the transcriptional target of both p53 and TAp63, the mRNA expression level of Bax followed a similar pattern to that of p53—induction 3.64-fold by DOX, 1.54-fold by the mimic, and suppression 0.34-fold by the inhibitor. In combination assays, Bax was reduced 0.83-fold with the inhibitor and slightly increased 1.28-fold with the mimic. Together, these results indicate that miR-203a-3p modulates DOX resistance through regulation of the p53 family.

### 3.4 MiR-203a-3p modulates DOX resistance through the Δ133p53 isoform in HepG2 cells

**FIGURE 4:**
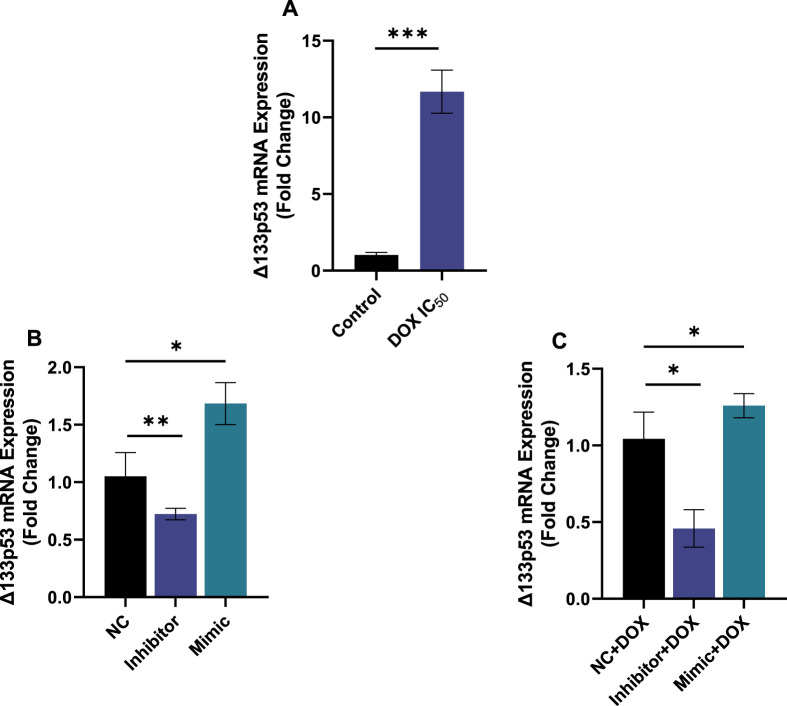
MiR-203a-3p modulates DOX resistance through the Δ133p53 isoform in HepG2 Cells. Effect of 2 µM of DOX **(A)**, 100 nM inhibitor and 50 nM mimic **(B)**, and combinations **(C)** on Δ133p53 expression in HepG2 cells detected by qPCR. The results were quantified by comparing to β-actin reference gene. Data are expressed as mean ± SEM (n = 4). Student’s t-test calculated statistical significance. *P < 0.05, **P < 0.01, ***P < 0.001 vs control, NC, and NC + DOX.

To further investigate the role of p53 and assess whether Δ133p53 contributes to miR-203a-3p–mediated DOX resistance in HepG2 cells, its expression was quantified by qPCR. DOX strongly induced Δ133p53 by 11.67-fold, whereas the inhibitor suppressed it by 0.72-fold and the mimic increased it by 1.68-fold relative to controls. In combination assays, the inhibitor reduced Δ133p53 by 0.46-fold, while the mimic enhanced it by 1.26-fold compared with the DOX-treated negative control (Figures 4A–C). These findings identify Δ133p53 as a DOX-inducible isoform regulated by miR-203a-3p, implicating its modulation in DOX resistance in HCC.

### 3.5 MiR-203a-3p modulates DOX resistance through TAp63 and Bax in Huh7 cells

**FIGURE 5:**
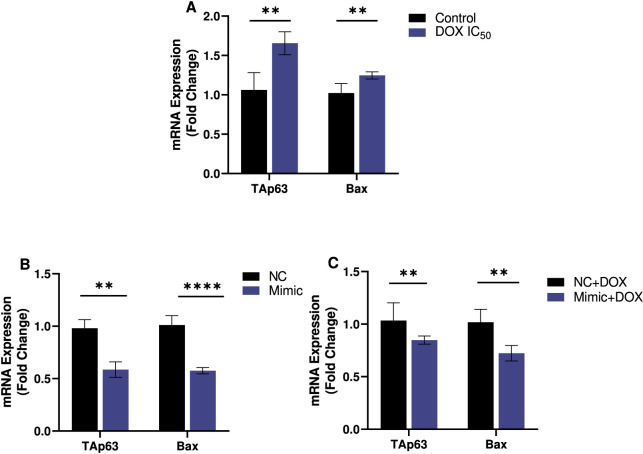
MiR-203a-3p modulates DOX resistance through TAp63 and Bax in Huh7 cells. Effect of 0.5 µM of DOX **(A)**, 30 nM mimic **(B)**, and combinations **(C)** on TAp63 and Bax expression in Huh7 cells detected by qPCR. The results were quantified by comparing to β-actin reference gene. Data are expressed as mean ± SEM (n = 4). Student’s t-test calculated statistical significance. *P < 0.05, **P < 0.01, ***P < 0.001 vs control, NC, and NC + DOX.

To assess the role of miR-203a-3p–mediated DOX resistance in the absence of functional p53, qPCR was used to measure TAp63 and Bax in Huh7 cells. As shown in Figures 5A–C, DOX increased TAp63 expression by 1.65-fold and Bax by 1.25-fold, whereas the miR-203a-3p mimic reduced both by 0.59- and 0.58-fold, respectively, relative to controls. In combination assays, the mimic suppressed TAp63 by 0.85-fold and Bax by 0.72-fold relative to DOX-treated negative control. These findings indicate that in p53-mutant Huh7 cells, TAp63 and Bax remain stress-inducible but are repressed by miR-203a-3p, implicating miR-203a-3p in DOX resistance.

### 3.6 MiR-203a-3p modulates DOX-induced apoptosis in HCC cells

**FIGURE 6:**
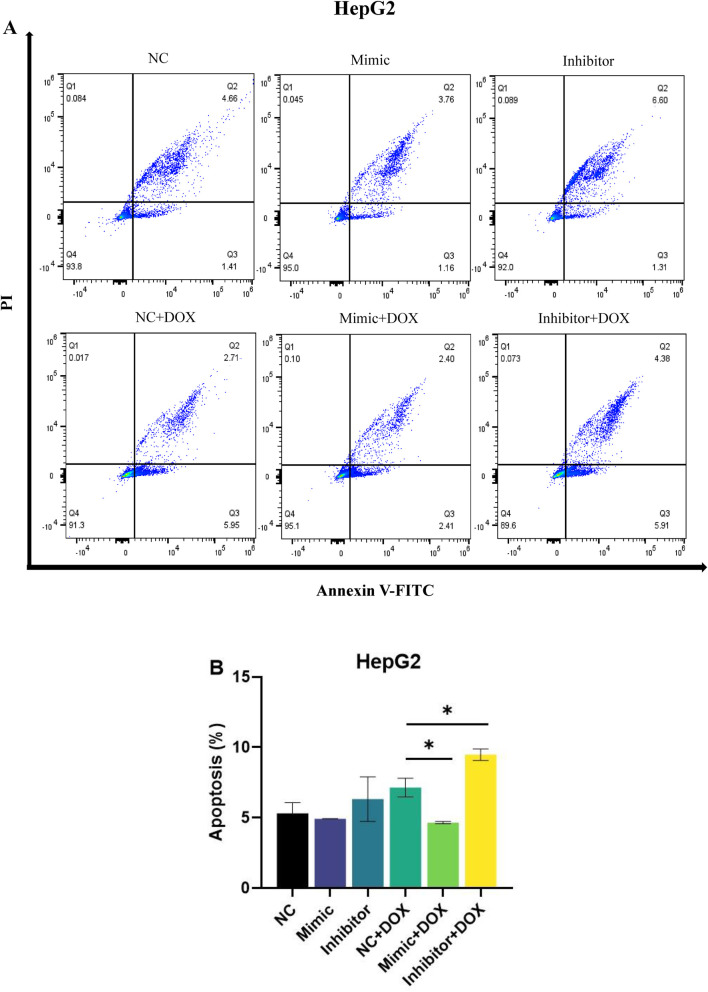
MiR-203a-3p modulates DOX-induced apoptosis in HepG2 cells. Cells transfected with 100 nM NC, 50 nM miR-203a-3p mimic, or 100 nM miR-203a-3p inhibitor were treated with 2 µM of DOX for 24 h, collected and stained with AnnexinV/PI for apoptotic analysis. Lower left quadrant indicates viable cells, lower right quadrant indicates early apoptotic cells, upper right quadrant indicates late apoptotic cells, upper left quadrant is necrotic cells **(A)**. Total apoptosis percentage is presented as early apoptosis + late apoptosis **(B)**. Data are expressed as mean ± SEM (n = 3). Statistical significance was calculated by one-way ANOVA followed by Tukey’s multiple comparisons test. *P < 0.05 vs NC + DOX.

**FIGURE 7:**
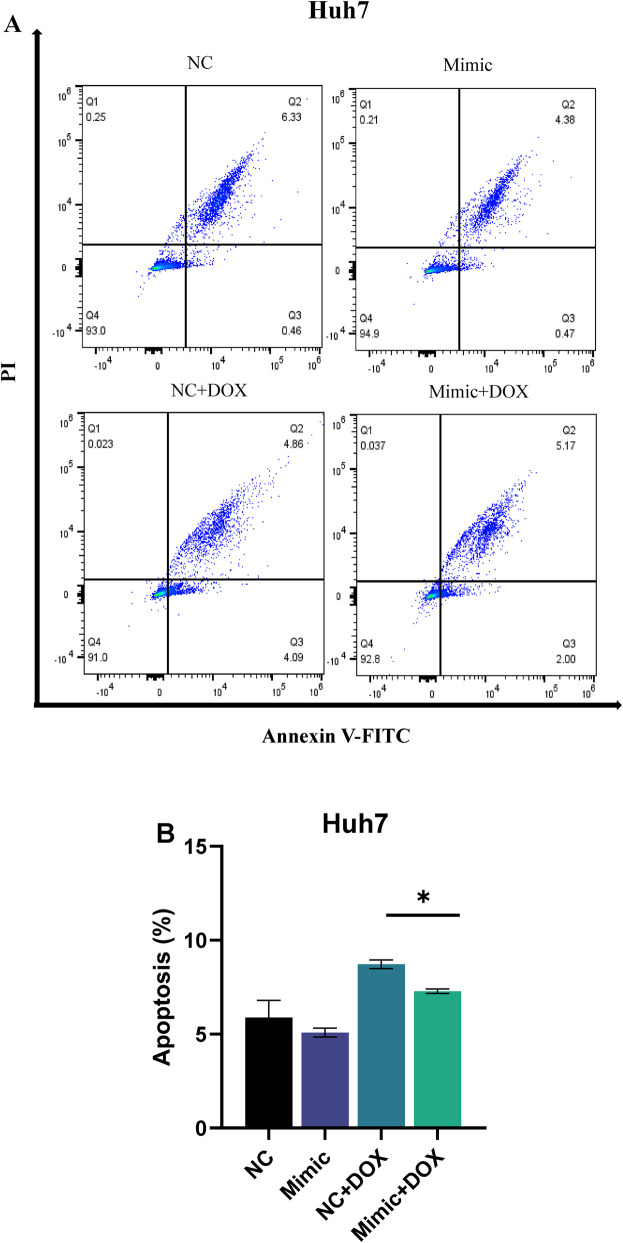
MiR-203a-3p modulates DOX-induced apoptosis in Huh7 cells. Cells transfected with 30 nM NC or 30 nM miR-203a-3p mimic were treated with 0.5 µM of DOX for 24 h, collected and stained with AnnexinV/PI for apoptotic analysis. Lower left quadrant indicates viable cells, lower right quadrant indicates early apoptotic cells, upper right quadrant indicates late apoptotic cells, upper left quadrant is necrotic cells **(A)**. Total apoptosis percentage is presented as early apoptosis + late apoptosis **(B)**. Data are expressed as mean ± SEM (n = 3). Statistical significance was calculated by one-way ANOVA followed by Tukey’s multiple comparisons test. *P < 0.05 vs NC + DOX.

To further determine the functional role of miR-203a-3p in mediating DOX resistance, apoptosis was assessed by Annexin V/PI staining. In HepG2 cells, inhibitor transfection alone slightly reduced viability to 92% compared with 93.8% in the negative control, accompanied by an increase in late apoptosis to 6.6% compared with 4.7%. In combination assays, viability decreased to 89.6% compared with 91.3% and late apoptosis increased to 4.4% compared with 2.7% in the DOX-treated negative control. Mimic transfection maintained high viability at approximately 95% with minimal apoptosis, and co-treatment with DOX did not increase apoptosis beyond that observed with the mimic alone (Figures 6A,B). In Huh7 cells, mimic transfection alone had little effect on apoptosis relative to the negative control. In combination assays, viability increased slightly to 92.8% and late apoptosis to 5.2%, compared with 91% viability and 4.9% late apoptosis in DOX-treated negative control. (Figures 7A,B). Overall, the results support a role for miR-203a-3p in regulating DOX-induced apoptosis in HCC cells.

### 3.7 MiR-203a-3p modulates the metabolic response to DOX in HCC cells

**FIGURE 8:**
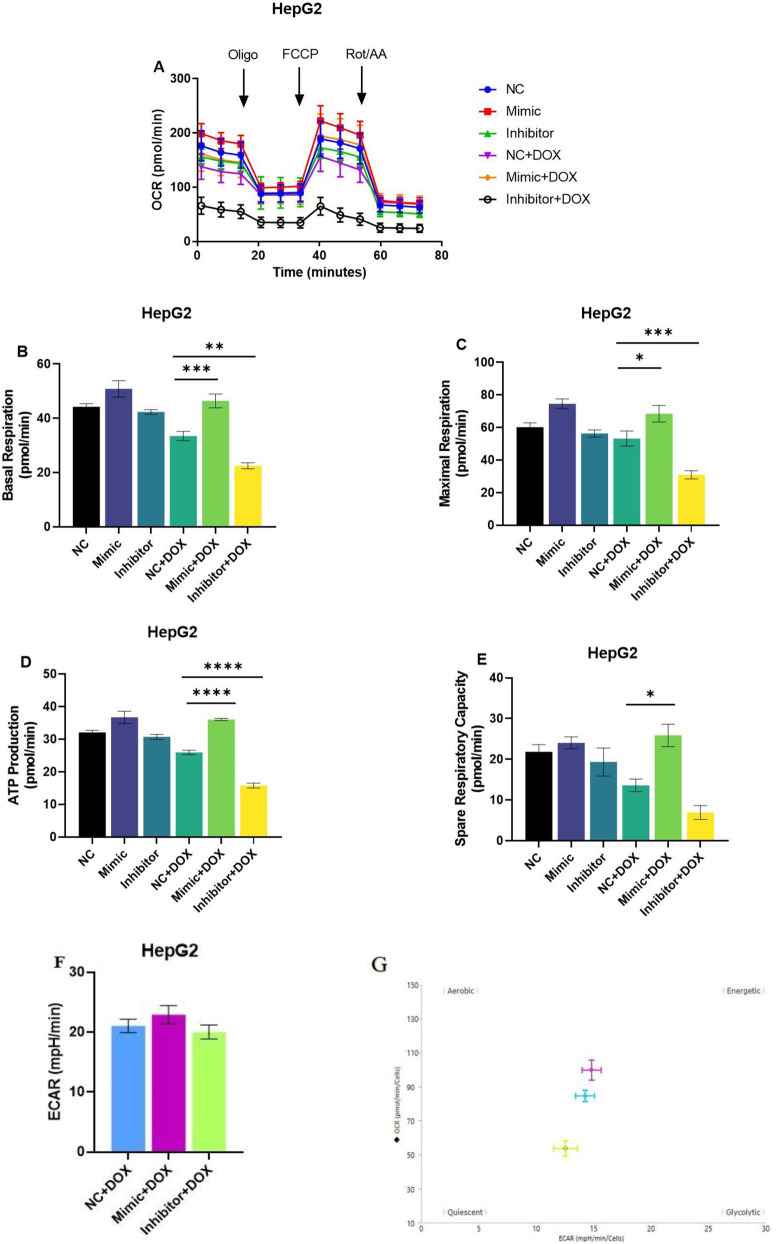
MiR-203a-3p modulates the metabolic response to DOX in HepG2 cells. Cells transfected with 100 nM NC, 50 nM miR-203a-3p mimic, or 100 nM miR-203a-3p inhibitor were treated with 2 µM of DOX for 24 h and OCR **(A)**, basal respiration **(B)**, maximal respiration **(C)**, ATP production **(D)**, spare respiratory capacity **(E)**, ECAR **(F)**, and overall bioenergetic capacity **(G)** were evaluated using the Seahorse XF Cell Mito Stress Test. Data are expressed as mean ± SEM (n = 6). Statistical significance was calculated by one-way ANOVA followed by Tukey’s multiple comparisons test. *P < 0.05, **P < 0.01, ***P < 0.001, ****P < 0.0001 vs NC + DOX.

**FIGURE 9:**
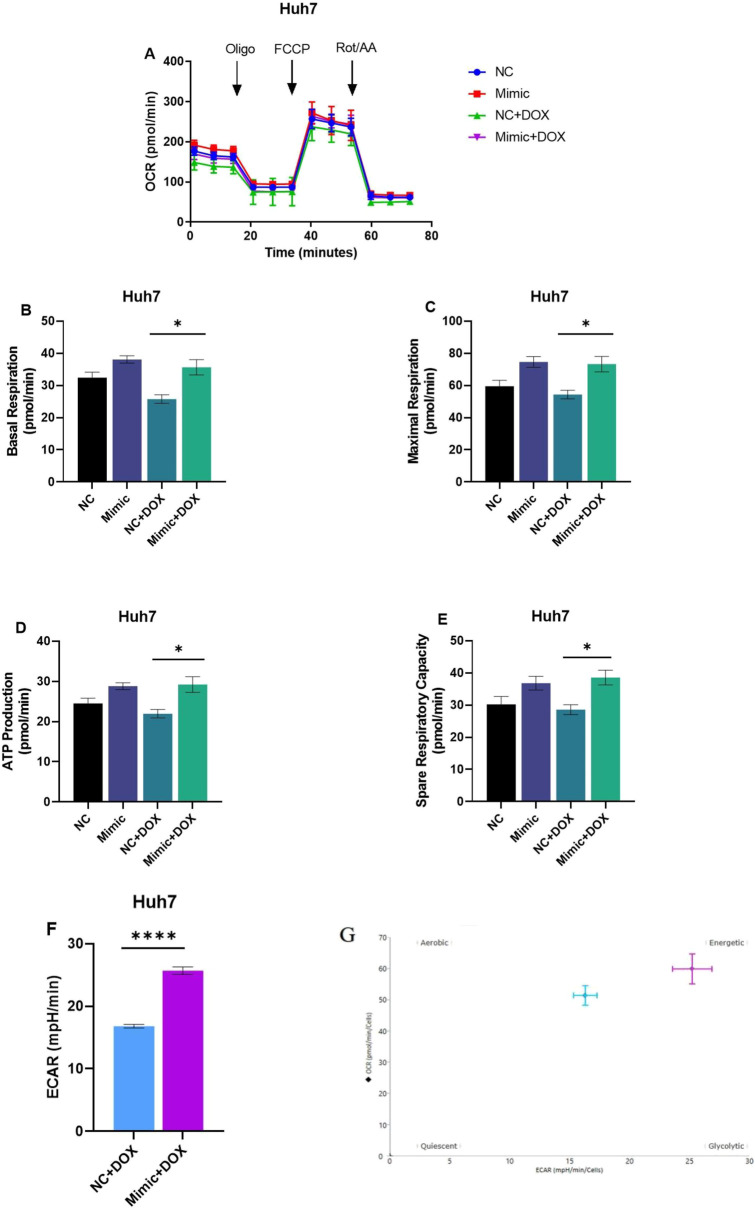
MiR-203a-3p modulates the metabolic response to DOX in Huh7 cells. Cells transfected with 30 nM NC or 30 nM miR-203a-3p mimic were treated with 0.5 µM of DOX for 24 h and OCR **(A)**, basal respiration **(B)**, maximal respiration **(C)**, ATP production **(D)**, spare respiratory capacity **(E)**, ECAR **(F)**, and overall bioenergetic capacity **(G)** were evaluated using the Seahorse XF Cell Mito Stress Test. Data are expressed as mean ± SEM (n = 6). Statistical significance was calculated by one-way ANOVA followed by Tukey’s multiple comparisons test. *P < 0.05, ****P < 0.0001 vs NC + DOX.

To investigate the role of miR-203a-3p in regulating the bioenergetic profile of HCC cells in response to DOX, mitochondrial function was assessed using the Seahorse XF Cell Mito Stress Test. In HepG2 cells, inhibitor transfection reduced mitochondrial activity, with decreases of 4%–6% in basal and maximal respiration and 4%–11% in ATP production and spare respiratory capacity relative to the negative control. In combination assays, basal and maximal respiration, and ATP production were reduced by 33%–41%, while spare capacity declined by 49% without significance compared with DOX-treated negative control. ECAR was unchanged, indicating limited impact on glycolysis. In contrast, mimic transfection enhanced mitochondrial activity, with 15%–24% increases in basal and maximal respiration and 10%–15% increases in ATP production and spare capacity. In combination assays, increases of 29%–90% across all mitochondrial parameters were observed. ECAR rose by 13% without significant glycolytic activation (Figures 8A–G). In Huh7 cells, mimic transfection also promoted mitochondrial activity, increasing basal and maximal respiration by 17%–25% and ATP production and spare capacity by 18%–22%. In combination assays, increases of 33%–39% across all mitochondrial parameters were observed. Unlike HepG2, Huh7 cells also showed a significant 53% rise in ECAR, indicating a combined upregulation of oxidative phosphorylation and glycolysis (Figures 9A–G). Overall, these results demonstrate that miR-203a-3p modulates cellular energy metabolism in HCC cells.

## 4 Discussion

Chemotherapeutic drug resistance is a significant challenge in cancer treatment, accounting for nearly 90% of cancer-related deaths (Wang et al., 2019). Combining miRNAs with chemotherapy enhances its efficacy and mitigates resistance and recurrence; therefore, identifying drug resistance-associated miRNAs is a critical area of research (Jin et al., 2017; Qiu et al., 2023; Wang Z. et al., 2024; Zhang et al., 2025). In this study, we investigated the role of miR-203a-3p in modulating DOX resistance and its relationship with the p53 family in HCC.

Consistent with previous reports, we found that HepG2 cells were more resistant to DOX than Huh7 cells, suggesting that inherent differences in p53 status may underlie the differential drug response observed between the two cell lines and provide a rationale for investigating the molecular determinants of DOX resistance in HCC (Hu et al., 2015; Kaplan, 2023; Lee et al., 2002). Basal miR-203a-3p levels were significantly higher in HepG2 cells relative to Huh7 cells, consistent with findings by Guo et al., suggesting a potential role in regulating DOX resistance in HCC (Guo et al., 2023). This difference is likely driven by the p53 status, as wild-type p53 was reported to enhance the post-transcriptional maturation of several miRNAs, including miR-203a-3p, through Drosha-dependent processing (Suzuki et al., 2009; Chang et al., 2013). Viability assays revealed that inhibition of miR-203a-3p sensitized HepG2 cells to DOX, while its overexpression enhanced resistance in both HepG2 and Huh7 cells. Although absolute IC_50_ values remained lower, Huh7 cells exhibited a greater fold increase in DOX resistance compared with HepG2 cells, indicating a stronger proportional effect. These results support a role for miR-203a-3p as a key regulator of DOX response in HCC.

Our findings align with previous studies in other cancers, including work by Zhou et al., who demonstrated that miR-203a-3p drives oxaliplatin resistance in colorectal cancer by targeting ataxia-telangiectasia mutated (ATM) and dampening DNA damage response pathways (Zhou et al., 2014). Similarly, Ru et al. reported that miR-203a-3p confers cisplatin resistance in breast cancer by targeting suppressor of cytokine signaling 3 (SOCS3) and suppressing p53-dependent pro-apoptotic signaling (Ru et al., 2011).

Mechanistically, our data reveal a complex, context-dependent regulatory network involving miR-203a-3p and members of the p53 family, providing potential molecular insights into the paradoxical chemoresistance observed in HepG2 cells harboring wild-type p53. We report a 7.91-fold increase in p53 expression, reflecting the acute activation of its signaling in response to DOX. Although p53 typically functions as a tumor suppressor that induces apoptosis in response to DNA damage, our results show that overexpression of miR-203a-3p leads to a parallel increase in the expression of p53, Δ133p53, and Bax, accompanied by downregulation of TAp63. This suggests a coordinated regulatory mechanism to promote cell survival and inhibit apoptosis, potentially through inducing cell cycle arrest and DNA damage repair.

Conversely, inhibition of miR-203a-3p reduces the expression of p53, Δ133p53, and Bax, while increasing TAp63. TAp63 functions as a potent inducer of apoptosis by directly transactivating multiple pro-apoptotic genes, including Bax. However, in HepG2 cells, our data indicate that Bax expression remains primarily under the control of wild-type p53. This suggests that elevated TAp63 expression inhibits cell survival and promotes apoptosis through alternative downstream targets, potentially involving other pro-apoptotic genes and the activation of death receptor-mediated apoptotic pathways. Collectively, these findings warrant further mechanistic investigations to confirm the direct target binding interactions mediated by miR-203a-3p.

Our findings are consistent with growing evidence that wild-type p53 can paradoxically promote treatment resistance by inducing cell cycle arrest and targeting Δ133p53, which enhances DNA repair, inhibits apoptosis, and supports survival under genotoxic stress (Gong et al., 2015; Gong et al., 2016; Jackson et al., 2012; Tomas et al., 2024; Ungerleider et al., 2018; Webster et al., 2020). They are also consistent with emerging data implicating Bax, a well-characterized pro-apoptotic gene, in the development of chemoresistance, including research by Funk et al. which revealed that during hepatocarcinogenesis, altered Bax localization independent of transcriptional regulation may represent a novel mechanism for evading apoptosis and acquiring resistance (Funk et al., 2020; Kale et al., 2018; Wang S. et al., 2024). As well as findings that suppression of TAp63 promotes HCC cell proliferation and survival (Gu et al., 2018; Xie et al., 2022).

In Huh7 cells, DOX induced TAp63 and Bax, suggesting that TAp63 compensates for the loss of wild-type p53. Overexpression of miR-203a-3p suppressed both, reducing apoptotic signaling and promoting resistance. These findings are consistent with our proposed inhibitory role of miR-203a-3p on TAp63 expression. Overall, the results suggest that, in the absence of wild-type p53, miR-203a-3p contributes to DOX resistance by suppressing the pro-apoptotic TAp63/Bax axis. Our findings are supported by Yao and Chen, who demonstrated that TAp63 is essential for DOX-induced apoptosis in p53-deficient HCC cells and by Gunaratne et al. who reported that TAp63 activity enhances sensitivity to cisplatin in p53-mutant ovarian cancer (Gunaratne et al., 2019; Yao and Chen, 2010).

Apoptosis assays confirmed these molecular observations. Inhibition of miR-203a-3p enhanced DOX-induced apoptosis in HepG2 cells, while its overexpression reduced apoptosis in both cell lines. Collectively, these results identify miR-203a-3p as an anti-apoptotic miRNA that attenuates the cytotoxic effects of DOX in HCC, independent of p53 status. Notably, the larger effect size observed in the MTT-derived IC_50_ values compared with the Annexin V/PI apoptosis readout likely reflects the fact that miR-203a-3p regulates multiple determinants of DOX response beyond apoptosis alone. While Annexin V/PI specifically captures apoptotic cells at 24 h, the MTT assay reflects overall viable metabolic activity and is strongly influenced by mitochondrial function and proliferative capacity. This distinction is particularly relevant in our study because miR-203a-3p overexpression markedly enhanced oxidative phosphorylation in both HepG2 and Huh7 cells and additionally increased glycolytic activity in Huh7 cells. Therefore, the relatively modest changes in apoptosis are consistent with a broader chemoresistant phenotype in which miR-203a-3p promotes survival not only by attenuating apoptotic signaling but also by promoting metabolic adaptation that sustains cell viability under DOX stress.

Beyond apoptosis, we investigated cellular metabolism, since metabolic plasticity is increasingly recognized as a hallmark of drug resistance (Ma and Zong, 2020). In HepG2 cells, miR-203a-3p inhibition impaired mitochondrial respiration, reducing basal and maximal respiration, ATP production, and spare capacity. Conversely, its overexpression enhanced oxidative phosphorylation with minimal effects on glycolysis, suggesting that miR-203a-3p promotes resistance by inducing a preferential shift toward oxidative phosphorylation rather than a global upregulation of energy metabolism pathways. In Huh7 cells, miR-203a-3p overexpression not only enhanced oxidative phosphorylation but also increased glycolysis. This dual metabolic activation reflects metabolic plasticity, enabling cells to adapt more flexibly to therapeutic stress.

Our findings support a role for miR-203a-3p as a regulator of cellular metabolism in HCC in response to DOX and are consistent with studies showing its role in metabolic rewiring in ovarian and esophageal cancers (Liu et al., 2020; Xiaohong et al., 2016). Chang et al. further demonstrated that miR-203a-3p sensitizes melanoma cells to temozolomide by targeting glutaminase-mediated glutamine metabolism (Chang et al., 2017). Additionally, reports across different cancer types align with our findings and highlight the diversity of metabolic responses adopted to overcome therapeutic stress. For instance, chemoresistant ovarian cancer cells increase mitochondrial energy production without a corresponding rise in glycolytic activity, whereas glioma cells exhibit a hyper-energetic phenotype, with simultaneous increases in glycolysis and mitochondrial respiration to sustain survival under therapeutic stress (Giddings et al., 2021; Rai et al., 2025).

The differential responses between HepG2 and Huh7 cells highlight the heterogeneity of HCC metabolism. In HepG2 cells, miR-203a-3p overexpression upregulated p53, which is known to enhance mitochondrial respiration by activating genes involved in controlling mitochondrial quality and respiration, while suppressing glycolysis through the inhibition of glucose transporters and glycolytic enzymes (Kitamura et al., 2011; Kondoh et al., 2005; Matoba et al., 2006; Schwartzenberg-Bar-Yoseph et al., 2004; Stambolsky et al., 2006). This p53-driven program favors oxidative metabolism and likely accounts for the observed increase in oxidative phosphorylation. The modest glycolytic response observed can be attributed to TAp63, which, similar to wild-type p53, promotes oxidative phosphorylation while suppressing glycolysis (Arianna et al., 2013; Venkatanarayan et al., 2015).

In Huh7 cells, harboring mutant p53, metabolic regulation may rely on other p53 family members such as TAp63. This is partly consistent with our findings, as miR-203a-3p overexpression led to TAp63 downregulation, which was accompanied by increased glycolytic activity. However, the expected suppression of oxidative phosphorylation was not observed. This suggests that miR-203a-3p may regulate metabolic pathways independently of TAp63, modulating mitochondrial function and oxidative phosphorylation through alternative metabolic targets.

## 5 Conclusion

Our study uncovers a novel mechanism by which miR-203a-3p enhances DOX resistance through suppressing apoptotic signaling and promoting metabolic plasticity. These findings carry significant clinical implications for the management of HCC. MiR-203a-3p holds potential as a predictive biomarker of DOX resistance, enabling early identification of patients unlikely to respond to treatment and guiding the use of alternative or combination therapies. Therapeutically targeting miR-203a-3p could restore chemosensitivity, potentially enhance the therapeutic efficacy of DOX while limiting systemic toxicity and improving patient outcomes. The underlying p53 family-dependent mechanism provides further therapeutic opportunities, including the use of pathway-specific agents to complement miR-203a-3p inhibition. Previous research has shown that miR-203a-3p contributes to chemoresistance in other cancers by regulating the cell cycle and apoptosis. Our study extends these observations to HCC and highlights a p53 family-dependent mechanism underlying miR-203a-3p-mediated drug resistance. Future work should explore the *in vivo* relevance of these findings and assess the therapeutic potential of miR-203a-3p inhibition in combination with other chemotherapeutic drugs.

## Data Availability

The original contributions presented in the study are included in the article/Supplementary Material, further inquiries can be directed to the corresponding author.
