# Supplementary material for: Regulation of doxorubicin resistance and cellular metabolism by miR-203a-3p via p53 and TAp63 signaling in hepatocellular carcinoma
Source: Front Pharmacol. 2026 Jun 12;17:1803189. doi: 10.3389/fphar.2026.1803189 (PMC13299100; doi:10.3389/fphar.2026.1803189)
Supplement: Supplementary file 1 [file Supplementaryfile1.pdf]

Supplementary tables

Table S1 | Primer sequences for qPCR-based detection of mRNAs.

**Target**	**Primer Sequences (5'-3')**
p53	F: CCCTTCCCAGAAAACCTACC<br>R: CTCCGTCATGTGCTGTGACT
TAp63	F: GACCTGAGTGACCCCATGTG<br>R: CGGGTGATGGAGAGAGAGCA
Bax	F: AAGCTGAGCGAGTGTCTCAAG<br>R: CAAAGTAGAAAAGGGCGACAAC
Δ133p53	F: ACTCTGTCTCCTTCCTCTTCCTACAG<br>R: GTGTGGAATCAACCCACAGCT
β-actin	F: AGCACAGAGCCTCGCCTTT<br>R: CACGATGGAGGGGAAGAC

Table S2 | miRCURY LNA miRNA primers for qPCR-based detection of miRNAs.

**Target**	**MiRNA Sequence (5'-3')**	**Assay ID**
hsa-miR-203a-3p	GUGAAAUGUUUAGGACCACUAG	YP00205914
hsa-miR-103a-3p	AGCAGCAUUGUACAGGGCUAUGA	YP00204063
